# High-grade fetal adenocarcinoma of the lung with abnormal expression of alpha-fetoprotein in a female patient: Case report

**DOI:** 10.1097/MD.0000000000024634

**Published:** 2021-02-19

**Authors:** Lu Xiao-Feng, Zhou Guo-Qi, Hu Wei, Li Jing-Hong, Ding Chao-Xia, Cai Xiao-Yan, Xun Yang

**Affiliations:** a Department of Respiratory and Critical Care Medicine, People's Hospital of Honghuagang District; b Department of Oncology, Second Affiliated Hospital of Medical University, Zunyi, China.

**Keywords:** alpha-fetoprotein, case report, fetal adenocarcinoma of the lung, high-level fetal adenocarcinoma of the lung, lung cancer

## Abstract

**Introduction:**

Fetal adenocarcinoma of the lung (FLAC) is an extremely rare tumor. Due to its rarity, most of the knowledge about FLAC comes from case reports. FLAC is an invasive adenocarcinoma that is similar to the fetal lung in the pseudo-glandular stage (8–16 weeks of gestation). Owing to the differences in histopathology and clinical process, FLAC has been further divided into low-level (L-FLAC) and high-level (H-FLAC). H-FLAC is usually associated with other conventional types of lung adenocarcinoma. Lung adenocarcinoma that produces alpha-fetoprotein (AFP) is a rare type of lung cancer. Its characteristics have not been fully elucidated.

**Patient's concerns:**

We recently encountered this type of FLAC in a 51-year-old female patient. A computed tomography (CT) scan of the chest revealed a 74 × 51-mm sized tumor in the lingual segment of the superior lobe of the left lung. Among the tumor markers, serum AFP was elevated (816.2 ng/mL).

**Primary diagnosis, interventions, and outcomes:**

The diagnosis of FLAC in this patient was confirmed by bronchoscopy with lung biopsy. Through a thoracoscope, left lung pneumonectomy, and mediastinal lymph node dissection were performed. The postoperative pathological results were consistent with the preoperative diagnosis of H-FLAC. Western blotting showed the difference in the AFP expression between the normal lung tissue and the cancerous lung tissue. Eventually, the diagnosis was AFP-producing H-FLAC. Using an immunohistochemical marker for AFP, cancer cells were shown to express AFP, specifically in their nuclei. After the operation, the patient underwent conventional chemotherapy. Her serum AFP gradually decreased over the course of 2 weeks.

**Conclusion:**

Presently, specific tumor markers for the diagnosis of lung cancer have not been established. To the best of our knowledge, this is the first case of abnormal AFP expression in a patient with H-FLAC. It may provide a basis for the clinical diagnosis of H-FLAC, a rare tumor, and AFP may be considered as a specific tumor marker.

## 1 Introduction

Fetal adenocarcinoma of the lung (FLAC) is an extremely rare malignant lung tumor, which accounts for only 0.1%–0.5% of all primary lung tumors.^[1–3]^ In 2011, the new international multidisciplinary lung adenocarcinoma classification, jointly developed by the International Lung Cancer Research Association, the American Thoracic Society, and the European Respiratory Society, classified FLAC as a variant of invasive adenocarcinoma.^[4]^ FLAC is similar to a developing fetal lung during its pseudo-glandular phase (8–16 weeks of gestation).^[5–7]^ Although literature reports that FLAC presents with intrabronchial invasion, the tumor is usually solitary, well-defined, and located on the periphery.^[8]^ From a pathological perspective, FLAC contains complex branched tubular glands lined with nonciliated columnar or cuboidal cells, rich in glycogen. The cells have a clear cytoplasm. In addition to the supranuclear or subnuclear vacuoles, large vesicular nuclei can also be seen. The malignant glands are densely arranged and located in a loose to moderately loose cell fibroblast matrix.^[9,10]^ Poor prognostic factors for FLAC are thoracic lymphadenopathy, metastasis at diagnosis, and tumor recurrence. The 10-year survival rate of FLAC is approximately 75%.^[11]^

Alpha-fetoprotein (AFP) is a protein formed in the liver and yolk sac of the fetus. High AFP levels are initially found in human fetal serum, and the level of serum AFP decreases after birth. Therefore, elevated AFP levels in an adult is abnormal.^[12]^ Regarding the pathological significance of this protein in adults, serum AFP is usually elevated in patients with liver cancer or gonadal germ cell tumors (such as yolk sac tumors). Because serum AFP levels can reduce after effective treatment, it is of clinical value to measure serum AFP levels during patient follow-up or to detect tumor recurrence after treatment. There are reports on ovarian, gastric, and lung cancers that produce AFP.^[13]^ Therefore, we report a rare case of AFP-producing H-FLAC. This case report aims to search for specific tumor markers that will provide a basis for early diagnosis of lung cancer and improve the patient survival rate.

## 2 Patient information

A 51-year-old female patient presented with productive cough in October 2019 in our hospital. The patient reported no abnormal diet, normal bowel movement, and no significant weight loss. The patient had no history of dust exposure, no history of smoking or drinking, and no history of familial tumors or hereditary diseases.

## 3 Clinical findings

Her physical examination found that the breath sounds of both lungs were normal. There were also no abnormal findings in the heart and abdomen.

## 4 Timeline

## 5 Diagnostic assessment

**Figure 1:**
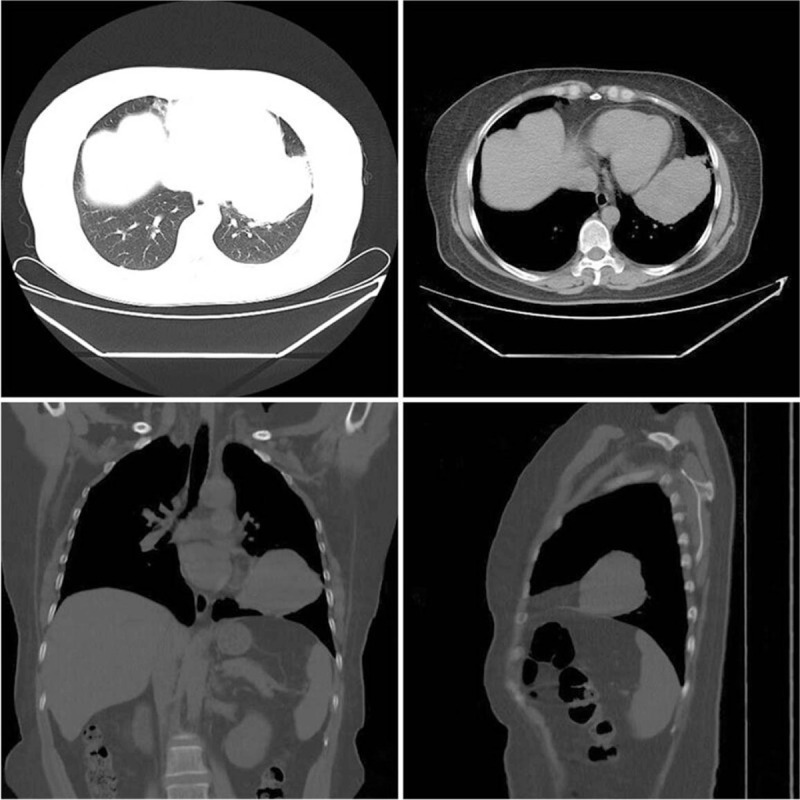
Chest computed tomography (CT) scan showing a soft tissue mass in the tongue segment of the left superior lobe of the left lung. Tumor size was approximately 74 × 51 mm, and the CT value was 33 HU. A small liquefaction component was seen in the soft tissue mass.

A chest computed tomography (CT) scan (Fig. 1) showed a lobular mass in the lingual segment of the left superior lobe of the left lung, with an approximate size of 74 × 51 mm. There were no clear enlarged lymph nodes bilaterally, in the hilum and mediastinum. Serum analysis showed significantly increased AFP values at 816.2 ng/mL (normal <5.0 ng/mL), whereas the remaining tumor markers were in the normal range. The diagnosis on admission was left lung mass with a high suspicion of malignancy. An electronic bronchoscopy showed inflammation in the bronchioles of the left superior lobe, inferior lobe, and tongue segment; compression of the openings of the lingula in the left superior lobe and inferior lobe of the left lung; and basal segment compression (no abnormal signal detected). A lung biopsy was performed to further confirm the diagnosis of primary bronchial lung cancer. Due to the lack of puncture tissue, the pathologist considered H-FLAC. The abdominal CT, head magnetic resonance imaging, and bone scan showed no distant metastasis. Based on the clinical and pathological findings, the mass had a grade of T4N0M0, making it stage IIIA.

## 6 Therapeutic intervention

**Figure 2:**
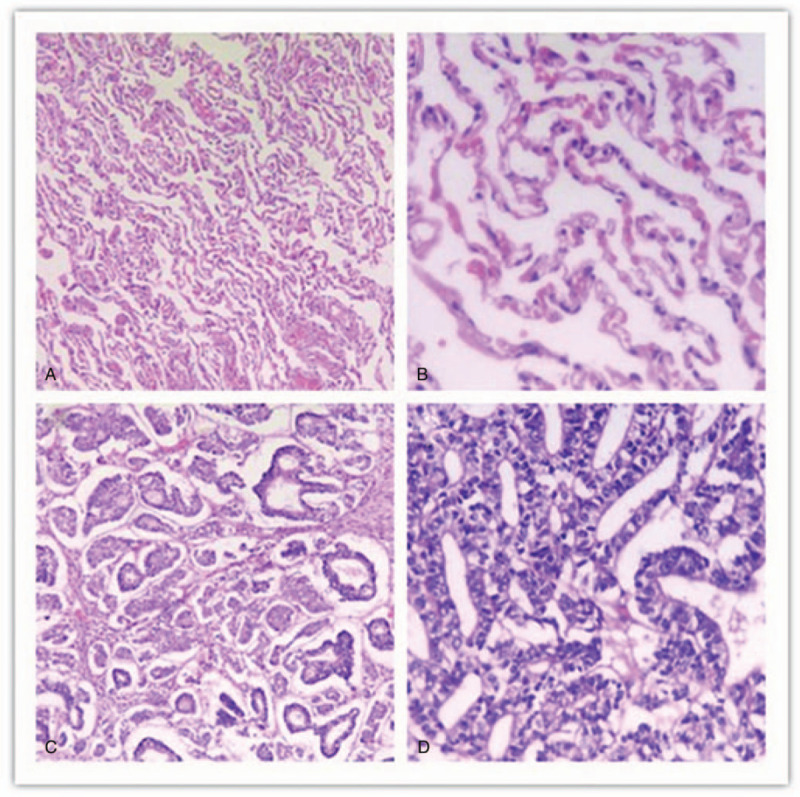
Hematoxylin and eosin staining of the normal lung tissue and affected lung tissue in patients with high-level fetal adenocarcinoma of the lung. (A) (magnification ×100) and (B) (magnification ×200) show the pathology of the normal lung tissue. (C) (magnification ×100) and (D) (magnification ×200) show the histopathological changes in the affected lung tissue. The tumor consists of complex glandular structures lined with glycogen-rich high columnar cells with obvious nuclear atypia and extensive necrosis.

**Figure 3:**
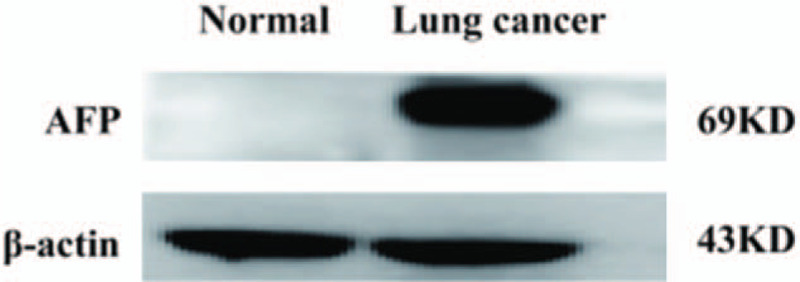
Levels of alpha-fetoprotein (AFP) in patients with high-level fetal adenocarcinoma of the lung (H-FLAC). Representative bands of AFP in the tumor tissue and normal lung tissue of a patient with H-FLAC. The grayscale values of AFP in the tumor and normal lung tissues were 20,727 ± 8955 ng/mL and 4733 ± 1247 ng/mL, respectively. AFP expression was significantly higher in the tumor tissue than that in the normal lung tissue, wherein AFP was hardly expressed.

**Figure 4:**
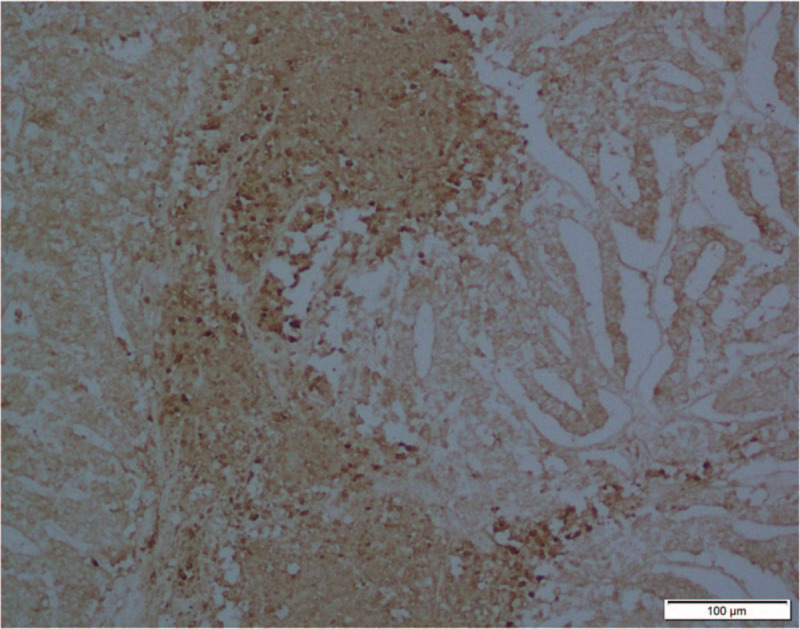
Immunohistochemistry of AFP in patients with high-level fetal adenocarcinoma of the lung. The arrangement of atypical cells is similar to that in fetal lungs in the pseudo-adenoid stage, with obvious nuclear atypia. Cancer cells express AFP, which is usually found in the nuclei. Bar = 100 μm. AFP = alpha-fetoprotein.

On November 6, 2019, through a thoracoscope, left lung pneumonectomy and mediastinal lymph node dissection were performed. An intraoperative exploration found that the left superior lobe of the left lung had a mass with a size of about 90 × 85 mm. The tumor invaded the left inferior lung lobe of the left lobe. The surgeon separated the adhesive tape and freely revealed the left superior lobe vein and left superior lobe artery with an endoscopic linear suture. Finally, the bronchus of the left lung was resected using an endoscopic linear suture device and then was removed from the chest wall incision. The lymph nodes in groups 5, 6, 7, 9, and 10 were resected. The postoperative pathological diagnosis (Fig. 2) was H-FLAC with a size of about 68 × 70 × 45 mm. The tumor involved the visceral pleura with intravascular infiltration, but no nerve infiltration. No tumor tissue was found at 4 cm from the incision margin of the lung parenchyma, 5 cm from the incision margin of the bronchus, and 4.4 cm from the incision margin of the blood vessel. The area of the group 5 lymph nodes was surrounded with adipose tissue, and no lymph nodes were seen. No tumor tissue was found in the lymph nodes of group 6 (0/5), group 7 (0/1), group 9 (0/1), and group 10 (0/1). Immunohistochemical staining results were as follows: TTF-1 (+), CK7 (+), β-catenin (+), Napsin-A (−), EMA (+), CEA (−), CD56 (−), SYN (−), Vim (−), and 34BE12 (−). Normal lung tissue and tumor tissue were tested for AFP antibody using a western blot (Fig. 3A) and then analyzed in parallel, as shown in Figure 3B. The results of the western blot of the normal lung tissue and cancerous lung tissue were different. Immunohistochemical staining was used to show the location of AFP in the cancerous cells, specifically in the nuclei (Fig. 4).

## 7 Follow-up and outcomes

The patient recovered without complications and underwent adjuvant chemotherapy. The chemotherapy regimen used was pemetrexed disodium 500 mg/kg and cisplatin 75 mg per cycle. Until May 2020, after 4 cycles of regular follow-up, no recurrence was observed.

## 8 Discussion

FLAC represents a rare subtype of lung neoplasia that harbors characteristics depending on the age of onset, which is a risk factor in itself.^[14]^ H-FLAC predominantly appears in elderly men, especially those over 50 years of age.^[9]^ Most patients with H-FLAC have no clinical symptoms, and most of them are found during physical examination or incidentally during a chest radiography. Its symptoms are related to bronchial irritation wherein chest pain is the typical manifestation. There are also reports of cough, hemoptysis, and dyspnea. Pleural effusion is rare.^[15,16]^ In this report, the patient mainly presented with cough, without chest pain and hemoptysis. The patient had no specific clinical symptoms, and this is consistent with the fact that H-FLAC usually presents with a more advanced-stage disease (stage III–IV).^[17]^ The 10-year survival for FLAC is approximately 75%.

Patients with H-FLAC usually present with a peripheral lesion with clear boundaries, whose size ranges from 2 to 12 cm.^[7]^ On CT, these tumors exhibit a heterogeneous appearance in which the threads of soft tissue are enhanced, and the necrotic areas are not enhanced. Metastases to the same or opposite lung or to the mediastinal compartment may also be apparent.^[18]^ As shown in Figure 1, a chest CT scan showed a soft tissue mass in the lingula of the left superior lobe of the left lung with a size of 74 × 51 mm. Its CT value was about 33 HU. A small liquefaction component was seen in the soft tissue mass, which is consistent with literature. The clinical findings of this patient, which were indications for surgery, have an important clinical value to the early diagnosis of H-FLAC.

FLAC typically presents with a lung that has a fetal morphology of at least 50%. It is usually associated with other conventional types of lung adenocarcinomas, such as squamous, papillary, and solid patterns. In addition to lacking morule formations, H-FLAC has prominent nuclear atypia, prominent nucleoli, and frequent mitosis.^[19]^ As shown in Figure 2, the tumor consists of complex glandular structures lined with glycogen-rich high columnar cells, with obvious nuclear atypia and large amounts of necrosis seen in hematoxylin and eosin-stained sections. These are typical manifestations of tumor cells and are clearly classified by immunohistochemistry. From a pathologic point of view, due to less puncture tissue in H-FLAC, it is difficult to diagnose the disease preoperatively. The diagnosis is usually made based on postoperative specimens or by autopsy.^[20]^ Therefore, although pathology is the gold standard for diagnosis of H-FLAC, there are still limitations.

It has been reported that patients with H-FLAC may present with elevated serum levels of AFP.^[21]^ AFP is one of the fetal proteins. Its molecular weight is intermediate between that of albumin and α1-globulin. The number of lung cancers that produce AFP has long been reported to be about 2% of all lung cancers.^[22]^ Histologically, adenocarcinomas, such as H-FLAC, account for most of the AFP-producing lung cancers. In Figure 3, we found that the level of AFP in patients with H-FLAC was significantly increased. This result was consistent with serological examination. As shown in Figure 4, by labeling AFP using immunohistochemistry in patients with H-FLAC, we found that the arrangement of atypical cells is similar to that in the fetal lungs in the pseudo-adenoid stage, with obvious nuclear atypia. Cancer cells express AFP, which is usually found in the nuclei. This result is consistent with the western blot findings. This finding has an important clinical significance, but it is rarely reported in literature. However, the serum levels of AFP returned to normal soon after tumor resection, and their pathological significance remained unknown.

Presently, specific tumor markers for the diagnosis of lung cancer have not been established. The diagnosis of special types of tumors, such as H-FLAC, is even more difficult. To the best of our knowledge, this is the first case of abnormal AFP expression in a patient with H-FLAC. It may provide a basis for the clinical diagnosis of H-FLAC, a rare tumor, and AFP may be considered as a specific tumor marker through this case.

## Acknowledgments

The authors are grateful to all the medical staff in our department.

## Author contributions

**Data curation:** Zhou Guo Qi, Hu Wei, Cai Xiao Yan, Xun Yang.

**Investigation:** Li Jing Hong, Ding Chao Xia.

**Writing – review & editing:** Lu Xiao Feng.
